# The Annual Report of the Eastern Asylum in the City of Williamsburg, Virginia, for 1844

**Published:** 1845-05

**Authors:** 


3. By the report of the Eastern Asylum we learn that the number of patients in that Institution on the 1st of January, 1844, was :—

| | Men. | Women. | Total. |
|---|---|---|---|
| | 62 | 47 | 109 |
| Received since, | 17 | 24 | 41 |
| Under care during the year, | 79 | 71 | 150 |
| Discharged during the year, | 7 | 5 | 12 |
| Died during the year, | 3 | 3 | 6 |
| Remaining December 31, | 69 | 63 | 132 |

A large proportion of the cases received in this Asylum have been chronic, and on that account, notwithstanding it is furnished with all the means and appliances of a successful curative establishment, the number of recoveries reported has been small.

Dr. Galt attempts to show the influence of the seasons in causing insanity, and concludes, from the statistics of the Eastern Asylum, that Summer is more productive of the disease than any of the other seasons. He also brings forward statistics of other Institutions, both in this country and Europe, in which the number of admissions during the six warm months, is much greater than in the remaining cold months of the year. It is evident, however, that inferences drawn from such data cannot be conclusive, inasmuch as they refer only to the time of admission, and not to that of the attack.

